# Supplementary material for: One size does not fit all: Nurturing identity needs and job satisfaction through employee benefits across gender and age
Source: PLoS One. 2025 Dec 10;20(12):e0336808. doi: 10.1371/journal.pone.0336808 (PMC12694837; doi:10.1371/journal.pone.0336808)
Supplement: S1 Text — (DOCX) [file pone.0336808.s001.docx]

# **S1 Text. Preliminary findings and ANOVA omnibus effect tables**

## **Study 1**

## **Preliminary findings**

We preliminarily explored whether there were differences related to gender and age in the quantitative and qualitative indicators of the different categories of benefits and employees’ identity needs. We performed different linear mixed models for each dependent variable utilising the GAMLj module in jamovi version 2.3.28.0 [70]. For each model, we included gender and age as fixed factors and the company ID as random factor.

### **Gender and age differences in the identity needs.**

The R^2^ Marginal of the final model was = .01 (R^2^ Conditional = .17). The random factor company ID ICC was .16. Table S1 reports the effects of the performed linear mixed model. Generally, women (M = 3.16, SD = .85) and employees under 50 (M = 3.30, SD = .87) showed lower identity needs fulfilment compared to men (M = 3.38, SD = .87) and employees over 50 (M = 3.35, SD = .87) respectively.

**Table S1. Linear Mixed Model Analysis for Identity Needs (N = 13368).**

|  | F | df | *p* |
| --- | --- | --- | --- |
| Gender | 120.25 | 1,13364 | < .001 |
| Age | 6.72 | 1,13365 | .01 |

**Gender and age differences in the quantitative indicator of the work-life integration benefits.** The R^2^ Marginal of the final model was = .03 (R^2^ Conditional = .11). The random factor company ID ICC was .09. Table S2 reports the effects of the performed linear mixed model. Generally, women (M = .16, SD = .40) and employees under 50 (M = .13, SD = .36) utilise more work-life integration benefits compared to men (M = .08, SD = .27) and employees over 50 (M = .07, SD = .26) respectively.

**Table S2. Linear Mixed Model Analysis for Utilisation of Work-life Integration Benefits (N = 13267).**

|  | F | df | *p* |
| --- | --- | --- | --- |
| Gender | 193 | 1,13264 | < .001 |
| Age | 144 | 1,13257 | < .001 |

**Gender and age differences in the qualitative indicator of the work-life integration benefits.**

The R^2^ Marginal of the final model was = .004 (R^2^ Conditional = .07). The random factor company ID ICC was .07. Table S3 reports the effects of the performed linear mixed model. Overall, employees under 50 (M = 3.80, SD = .86) are more satisfied with the work-life integration benefits they used compared to employees over 50 (M = 3.65, SD = .98).

**Table S3. Linear Mixed Model Analysis for Satisfaction with Work-life Integration Benefits (N = 1263).**

|  | F | df | *p* |
| --- | --- | --- | --- |
| Gender | .26 | 1,1260 | .61 |
| Age | 5.30 | 1,1259 | .02 |

**Gender and age differences in the quantitative indicator of the financial benefits.**

The R^2^ Marginal of the final model was = .001 (R^2^ Conditional = .40). The random factor company ID ICC was .40. Table S4 reports the effects of the performed linear mixed model. Employees under 50 (M = 1.12, SD = .86) utilise more financial benefits compared to employees over 50 (M = 1.12, SD = .85).

**Table S4. Linear Mixed Model Analysis for Utilisation of Financial Benefits (N = 13368).**

|  | F | df | *p* |
| --- | --- | --- | --- |
| Gender | 2.40 | 1,13362 | .12 |
| Age | 3.84 | 1,13363 | .05 |

**Gender and age differences in the qualitative indicator of the financial benefits.**

The R^2^ Marginal of the final model was = .001 (R^2^ Conditional = .15). The random factor company ID ICC was .15. Table S5 reports the effects of the performed linear mixed model. Overall, men (M = 3.44, SD = .81) are more satisfied with the financial benefits they used compared to women (M = 3.40, SD = .85).

**Table S5. Linear Mixed Model Analysis for Satisfaction with Financial Benefits (N = 9747).**

|  | F | df | *p* |
| --- | --- | --- | --- |
| Gender | 8.80 | 1,9743 | .003 |
| Age | .004 | 1,9744 | .99 |

**Gender and age differences in the quantitative indicator of the socio/cultural benefits.**

The R^2^ Marginal of the final model was = .004 (R^2^ Conditional = .50). The random factor company ID ICC was .50. Table S6 reports the effects of the performed linear mixed model. Generally, women (M = .67, SD = .81) and employees over 50 (M = .42, SD = .66) utilise more social and cultural benefits compared to men (M = .56, SD = .71) and employees under 50 (M = .38, SD = .64) respectively.

**Table S6. Linear Mixed Model Analysis for Utilisation of Socio/Cultural Benefits (N = 13368).**

|  | F | df | *p* |
| --- | --- | --- | --- |
| Gender | 101.93 | 1,13362 | < .001 |
| Age | 3.91 | 1,13363 | .05 |

**Gender and age differences in the qualitative indicator of the socio/cultural benefits.**

The R^2^ Marginal of the final model was = .01 (R^2^ Conditional = .20). The random factor company ID ICC was .19. Table S7 reports the effects of the performed linear mixed model. Overall, women (M = 3.14, SD = .97) are more satisfied with the social and cultural benefits they used compared to men (M = 2.89, SD = 1.00).

**Table S7. Linear Mixed Model Analysis for Satisfaction with Socio/Cultural Benefits (N = 6115).**

|  | F | df | *p* |
| --- | --- | --- | --- |
| Gender | 78.15 | 1,6112 | < .001 |
| Age | .60 | 1,6112 | .44 |

**Gender and age differences in the quantitative indicator of the health/safety benefits.**

The R^2^ Marginal of the final model was = .004 (R^2^ Conditional = .93). The random factor company ID ICC was .93. Table S8 reports the effects of the performed linear mixed model. Overall, women (M = .21, SD = .48) utilise more health/safety benefits compared to men (M = .05, SD = .34).

**Table S8. Linear Mixed Model Analysis for Utilisation of Health/Safety Benefits (N = 13368).**

|  | F | df | *p* |
| --- | --- | --- | --- |
| Gender | 742.73 | 1,13361 | < .001 |
| Age | .59 | 1,13361 | .44 |

**Gender and age differences in the qualitative indicator of the health/safety benefits.**

The R^2^ Marginal of the final model was = .002 (R^2^ Conditional = .09). The random factor company ID ICC was .09. Table S9 reports the effects of the performed linear mixed model. No significant difference between the groups emerged from the analysis.

**Table S9. Linear Mixed Model Analysis for Satisfaction with Health/Safety Benefits (N = 1058).**

|  | F | df | *p* |
| --- | --- | --- | --- |
| Gender | .01 | 1,1017 | .93 |
| Age | 1.89 | 1,1043 | .17 |

## **ANOVA omnibus effect tables**

**Table S10**. **Linear Mixed Model Analysis for Work-life Integration Benefits (N = 1263).**

|  | Model 1 | | | | Model 2 | | | | Model 3 | | | |
| --- | --- | --- | --- | --- | --- | --- | --- | --- | --- | --- | --- | --- |
|  | F | | df | *p* | F | | df | *p* | F | | df | *p* |
| Gender | 9.48 | | 1,1258 | .002 | 10.36 | | 1,1254 | .001 | 10.56 | | 1,1252 | .001 |
| Age | .04 | | 1,1258 | .83 | .02 | | 1,1253 | .89 | .08 | | 1,1251 | .78 |
| Utilisation Work-life Integration Benefits | .29 | | 1,1257 | .59 | 1.82 | | 1,1253 | .18 | 1.41 | | 1,1251 | .24 |
| Satisfaction Work-life Integration Benefits | 82.80 | | 1,1258 | < .001 | 80.09 | | 1,1254 | < .001 | 64.22 | | 1,1252 | < .001 |
| Utilisation Work-life Integration Benefits x Gender |  | |  |  | 1.10 | | 1,1253 | .30 | .37 | | 1,1251 | .54 |
| Utilisation Work-life Integration Benefits x Age |  | |  |  | 2.21 | | 1,1252 | .14 | .79 | | 1,1250 | .38 |
| Satisfaction Work-life Integration Benefits x Gender |  | |  |  | .36 | | 1,1254 | .55 | .11 | | 1,1252 | .74 |
| Satisfaction Work-life Integration Benefits x Age |  | |  |  | .83 | | 1,1254 | .55 | .66 | | 1,1251 | .42 |
| Utilisation Work-life Integration Benefits x Gender x Age |  | |  |  |  | |  |  | 1.56 | | 1,1251 | .21 |
| Satisfaction Work-life Integration Benefits x Gender x Age |  | |  |  |  | |  |  | .06 | | 1,1251 | .80 |
|  | | R^2^ Conditional = .14  R^2^ Marginal = .06  ICC = .08 | | | | R^2^ Conditional = .14  R^2^ Marginal = .07  ΔR^2^ = .004, *p* = .29  ICC = .08 | | | | R^2^ Conditional = .14  R^2^ Marginal = .07  ΔR^2^ = .002, *p* = .29  ICC = .08 | | |

**Table S11. Linear Mixed Model Analysis for Financial Benefits (N = 9747).**

|  | Model 1 | | | | Model 2 | | | | Model 3 | | | |
| --- | --- | --- | --- | --- | --- | --- | --- | --- | --- | --- | --- | --- |
|  | F | | df | *p* | F | | df | *p* | F | | df | *p* |
| Gender | 79.85 | | 1,9740 | < .001 | 80.06 | | 1,9736 | < .001 | 78.51 | | 1,9734 | < .001 |
| Age | 7.23 | | 1,9742 | .01 | 6.98 | | 1,9738 | .01 | 6.75 | | 1,9736 | .01 |
| Utilisation Financial Benefits | 12.45 | | 1,9703 | < .001 | 9.80 | | 1,9699 | .002 | 9.80 | | 1,9701 | .002 |
| Satisfaction Financial Benefits | 613.63 | | 1,9742 | < .001 | 488.10 | | 1,9737 | < .001 | 385.73 | | 1,9736 | < .001 |
| Utilisation Financial Benefits x Gender |  | |  |  | .06 | | 1,9736 | .80 | .06 | | 1,9733 | .81 |
| Utilisation Financial Benefits x Age |  | |  |  | 1.01 | | 1,9738 | 31 | .73 | | 1,9735 | .39 |
| Satisfaction Financial Benefits x Gender |  | |  |  | 3.78 | | 1,9736 | .31 | 5.51 | | 1,9734 | .02 |
| Satisfaction Financial Benefits x Age |  | |  |  | < .001 | | 1,9737 | .98 | .55 | | 1,9733 | .46 |
| Utilisation Financial Benefits x Gender x Age |  | |  |  |  | |  |  | .03 | | 1,9734 | .85 |
| Satisfaction Financial Benefits x Gender x Age |  | |  |  |  | |  |  | 1.78 | | 1,9733 | .18 |
|  | | R^2^ Conditional = .25  R^2^ Marginal = .06  ICC = .21 | | | | R^2^ Conditional = .25  R^2^ Marginal = .06  ΔR^2^ = .003, *p* < .001  ICC = .20 | | | | R^2^ Conditional = .25  R^2^ Marginal = .06  I ΔR^2^ = .002, *p* = .27  ICC = .20 | | |

**Table S12**. **Linear Mixed Model Analysis for Socio/Cultural Benefits (N = 6115).**

|  | Model 1 | | | | Model 2 | | | | Model 3 | | | |
| --- | --- | --- | --- | --- | --- | --- | --- | --- | --- | --- | --- | --- |
|  | F | | df | *p* | F | | df | *p* | F | | df | *p* |
| Gender | 99.88 | | 1,6110 | < .001 | 104.29 | | 1,6106 | < .001 | 103.45 | | 1,6104 | < .001 |
| Age | .49 | | 1,6110 | .49 | .43 | | 1,6106 | .51 | 1.13 | | 1,6104 | .29 |
| Utilisation Socio/Cultural Benefits | 5.33 | | 1,6107 | .02 | 6.90 | | 1,6103 | .01 | 1.97 | | 1,6100 | .16 |
| Satisfaction Socio/Cultural Benefits | 254.22 | | 1,6110 | < .001 | 234.88 | | 1,6105 | < .001 | 239.09 | | 1,6103 | < .001 |
| Utilisation Socio/Cultural Benefits x Gender |  | |  |  | 2.70 | | 1,6104 | .10 | .20 | | 1,6103 | .66 |
| Utilisation Socio/Cultural Benefits x Age |  | |  |  | .48 | | 1,6105 | .49 | .04 | | 1,6102 | .85 |
| Satisfaction Socio/Cultural Benefits x Gender |  | |  |  | 3.12 | | 1,6104 | .08 | 4.33 | | 1,6102 | .04 |
| Satisfaction Socio/Cultural Benefits x Age |  | |  |  | .08 | | 1,6104 | .78 | .70 | | 1,6103 | .40 |
| Utilisation Socio/Cultural Benefits x Gender x Age |  | |  |  |  | |  |  | 1.27 | | 1,6102 | .26 |
| Satisfaction Socio/Cultural Benefits x Gender x Age |  | |  |  |  | |  |  | 6.61 | | 1,6102 | .01 |
|  | | R^2^ Conditional = .19  R^2^ Marginal = .05  ICC = .15 | | | | R^2^ Conditional = .19  R^2^ Marginal = .05  ΔR^2^ = .001, *p* = .05  ICC = .15 | | | | R^2^ Conditional = .19  R^2^ Marginal = .05  ΔR^2^ = <.001, *p* = .72  ICC = .15 | | |

**Table S13**. **Linear Mixed Model Analysis for Health/Safety Benefits (N = 1058).**

|  | Model 1 | | | | Model 2 | | | | Model 3 | | | |
| --- | --- | --- | --- | --- | --- | --- | --- | --- | --- | --- | --- | --- |
|  | F | | df | *p* | F | | df | *p* | F | | df | *p* |
| Gender | 14.86 | | 1,1051 | < .001 | 15.42 | | 1,1045 | < .001 | 14.89 | | 1,1042 | < .001 |
| Age | .34 | | 1,1053 | .56 | .11 | | 1,1049 | .74 | .08 | | 1,1047 | .77 |
| Utilisation Health/Safety Benefits | .16 | | 1,864 | .69 | .69 | | 1,801 | .41 | .17 | | 1,810 | .68 |
| Satisfaction Health/Safety Benefits | 34.532 | | 1,1052 | < .001 | 34.73 | | 1,1049 | < .001 | 31.19 | | 1,1047 | < .001 |
| Utilisation Health/Safety Benefits x Gender |  | |  |  | .07 | | 1,1047 | .79 | 1.03 | | 1,1046 | .85 |
| Utilisation Health/Safety Benefits x Age |  | |  |  | 5.09 | | 1,1046 | .02 | 3.00 | | 1,1043 | .08 |
| Satisfaction Health/Safety Benefits x Gender |  | |  |  | 1.22 | | 1,1049 | .27 | 1.04 | | 1,1046 | .31 |
| Satisfaction Health/Safety Benefits x Age |  | |  |  | 2.30 | | 1,1046 | .13 | 1.75 | | 1,1045 | .19 |
| Utilisation Health/Safety Benefits x Gender x Age |  | |  |  |  | |  |  | .01 | | 1,1045 | .93 |
| Satisfaction Health/Safety Benefits x Gender x Age |  | |  |  |  | |  |  | .003 | | 1,1045 | .96 |
|  | | R^2^ Conditional = .25  R^2^ Marginal = .04  ICC = .22 | | | | R^2^ Conditional = .24  R^2^ Marginal = .05  ΔR^2^ = .01, *p* = .01  ICC = .20 | | | | R^2^ Conditional = .24  R^2^ Marginal = .05  ΔR^2^ = .01, *p* = .06  ICC = .20 | | |

## **Discussion**

The preliminary results indicated that, overall, women and employees under 50 were less satisfied with their identity needs. In terms of service utilisation, women reported higher use of all categories of benefits, except for financial benefits. Employees under 50 utilised work-life integration and financial benefits more, whereas those over 50 reported higher use of socio-cultural services. When considering satisfaction with benefits, employees under 50 were more satisfied with work-life integration benefits, men with financial services, and women with socio-cultural benefits.

## **Study 2**

## **Preliminary findings**

We preliminarily explored whether there were differences related to gender and age in the utilisation and satisfaction with the different categories of benefits and the identity needs. We performed different linear mixed models for each dependent variable utilising the GAMLj module in jamovi version 2.3.28.0 [70]. For each model, we included gender and age as fixed factors and the company ID as random factor

**Gender and age differences in the identity needs.**

The R^2^ Marginal of the final model was = .001 (R^2^ Conditional = .04). The random factor company ID ICC was .04. Table S10 reports the effects of the performed linear mixed model.

Generally, women (M = 3.16, SD = .82) showed lower identity needs fulfilment compared to men (M = 3.24, SD = .89).

**Table S14. Linear Mixed Model Analysis for Identity Needs (N = 6337).**

|  | F | df | *p* |
| --- | --- | --- | --- |
| Gender | 8.00 | 1,6334 | .01 |
| Age | .01 | 1,6331 | .91 |

**Gender and age differences in job satisfaction.**

The R^2^ Marginal of the final model was = .004 (R^2^ Conditional = .02). The random factor company ID ICC was .01. Table S11 reports the effects of the performed linear mixed model. Generally, women (M = 6.61, SD = 1.79) showed lower job satisfaction compared to men (M = 6.86, SD = 1.82).

**Table S15. Linear Mixed Model Analysis for Job Satisfaction (N = 6337).**

|  | F | df | *p* |
| --- | --- | --- | --- |
| Gender | 26.32 | 1,6308 | < .001 |
| Age | 1.02 | 1,6211 | .31 |

**Gender and age differences in the quantitative indicator of the work-life integration benefits.**

The R^2^ Marginal of the final model was = .01 (R^2^ Conditional = .27). The random factor company ID ICC was .26. Table S12 reports the effects of the performed linear mixed model. Generally, women (M = .88, SD = 1.14) and employees under 50 (M = .82, SD = 1.04) utilise more work-life integration benefits compared to men (M = .68, SD = .97) and employees over 50 (M = .73, SD = 1.12) respectively.

**Table S16. Linear Mixed Model Analysis for Utilisation of Work-life Integration Benefits (N = 6045).**

|  | F | df | *p* |
| --- | --- | --- | --- |
| Gender | 102.75 | 1,6041 | < .001 |
| Age | 8.37 | 1,6042 | .004 |

**Gender and age differences in the qualitative indicator of the work-life integration benefits.**

The R^2^ Marginal of the final model was = .001 (R^2^ Conditional = .03). The random factor company ID ICC was .03. Table S13 reports the effects of the performed linear mixed model.

No significant difference between the groups emerged from the analysis.

**Table S17. Linear Mixed Model Analysis for Satisfaction with Work-life Integration Benefits (N = 2803).**

|  | F | df | *p* |
| --- | --- | --- | --- |
| Gender | .15 | 1,2799 | .70 |
| Age | .26 | 1,2786 | .61 |

**Gender and age differences in the quantitative indicator of the financial benefits.**

The R^2^ Marginal of the final model was = .005 (R^2^ Conditional = .37). The random factor company ID ICC was .37. Table S14 reports the effects of the performed linear mixed model. Women (M = 3.46, SD = 1.76) utilise more financial benefits compared to men (M = 3.45, SD = 1.85).

**Table S18. Linear Mixed Model Analysis for Utilisation of Financial Benefits (N = 1502).**

|  | F | df | *p* |
| --- | --- | --- | --- |
| Gender | 9.10 | 1,1498 | .003 |
| Age | 1.79 | 1,1499 | .18 |

**Gender and age differences in the qualitative indicator of the financial benefits.**

The R^2^ Marginal of the final model was = .003 (R^2^ Conditional = .003). The random factor company ID ICC was < .001. Table S15 reports the effects of the performed linear mixed model. Overall, women (M = 3.69, SD = .85) are more satisfied with the financial benefits they used compared to men (M = 3.59, SD = .86).

**Table S19. Linear Mixed Model Analysis for Satisfaction with Financial Benefits (N = 1453).**

|  | F | df | *p* |
| --- | --- | --- | --- |
| Gender | 4.24 | 1,663 | .04 |
| Age | < .001 | 1,253 | .98 |

**Gender and age differences in the quantitative indicator of the socio/cultural benefits.**

The R^2^ Marginal of the final model was = .01 (R^2^ Conditional = .23). The random factor company ID ICC was .23. Table S16 reports the effects of the performed linear mixed model. Generally, women (M = .83, SD = .95) utilise more social and cultural benefits compared to men (M = .71, SD = .83).

**Table S20. Linear Mixed Model Analysis for Utilisation with Socio/Cultural Benefits (N = 1293).**

|  | F | df | *p* |
| --- | --- | --- | --- |
| Gender | 12.84 | 1,1290 | < .001 |
| Age | 1.49 | 1,1290 | .22 |

**Gender and age differences in the qualitative indicator of the socio/cultural benefits.**

The R^2^ Marginal of the final model was = .01 (R^2^ Conditional = .01). The random factor company ID ICC was < .001. Table S17 reports the effects of the performed linear mixed model. Overall, women (M = 3.58, SD = 3.99) are more satisfied with the social and cultural benefits they used compared to men (M = 2.74, SD = 3.25).

**Table S21. Linear Mixed Model Analysis for Satisfaction with Socio/Cultural Benefits (N = 1124).**

|  | F | df | *p* |
| --- | --- | --- | --- |
| Gender | 16.18 | 1,1221 | < .001 |
| Age | .75 | 1,1221 | .39 |

**Gender and age differences in the quantitative indicator of the health/safety benefits.**

The R^2^ Marginal of the final model was = .001 (R^2^ Conditional = .57). The random factor company ID ICC was .57. Table S18 reports the effects of the performed linear mixed model.

Overall, men (M = .29, SD = .66) and employees under 50 (M = .35, SD = .72) utilise more health/safety benefits compared to women (M = .26, SD = .62) and employees over 50 (M = .15, SD = .45).

**Table S22. Linear Mixed Model Analysis for Utilisation of Health/Safety Benefits (N = 6337).**

|  | F | df | *p* |
| --- | --- | --- | --- |
| Gender | 7.59 | 1,6332 | .01 |
| Age | 8.08 | 1,6332 | .004 |

**Gender and age differences in the qualitative indicator of the of health/safety benefits.**

The R^2^ Marginal of the final model was < .001 (R^2^ Conditional = .03). The random factor company ID ICC was .03. Table S19 reports the effects of the performed linear mixed model.

No significant difference between the groups emerged from the analysis.

**Table S23. Linear Mixed Model Analysis for Satisfaction with Health/Safety Benefits (N = 1273).**

|  | F | df | *p* |
| --- | --- | --- | --- |
| Gender | .31 | 1,1263 | .58 |
| Age | .27 | 1,1267 | .60 |

**ANOVA omnibus effect tables**

**Table S24**. **Linear Mixed Model Analysis for Work-life Integration Benefits (N = 2803).**

|  | Model 1 | | | | Model 2 | | | | Model 3 | | |
| --- | --- | --- | --- | --- | --- | --- | --- | --- | --- | --- | --- |
|  | F | | df | *p* | F | df | *p* | | F | df | *p* |
| Gender | 2.01 | | 1,765 | .16 | 2.01 | 1,767 | .16 | | 2.35 | 1,805 | .13 |
| Age | .89 | | 1,197 | .35 | 1.10 | 1,201 | .30 | | .95 | 1,216 | .33 |
| Utilisation Work-life Integration Benefits | 5.47 | | 1,2798 | .02 | 2.62 | 1,2793 | .11 | | 3.76 | 1,2792 | .05 |
| Satisfaction Work-life Integration Benefits | 152.68 | | 1,1863 | < .001 | 125.24 | 1,2074 | < .001 | | 121.73 | 1,2133 | < .001 |
| Utilisation Work-life Integration Benefits x Gender |  | |  |  | .01 | 1,2794 | .93 | | .01 | 1,2791 | .94 |
| Utilisation Work-life Integration Benefits x Age |  | |  |  | 9.61 | 1, 2793 | .002 | | 3.12 | 1,2792 | .32 |
| Satisfaction Work-life Integration Benefits x Gender |  | |  |  | 3.47 | 1,2793 | .06 | | 1.01 | 1,2791 | .08 |
| Satisfaction Work-life Integration Benefits x Age |  | |  |  | .11 | 1, 2793 | .74 | | < .001 | 1,2791 | .98 |
| Utilisation Work-life Integration Benefits x Gender x Age |  | |  |  |  |  |  | | .01 | 1,2792 | .94 |
| Satisfaction Work-life Integration Benefits x Gender x Age |  | |  |  |  |  |  | | 7.55 | 1,2791 | .01 |
|  | | R^2^ Conditional = .07  R^2^ Marginal = .05  ICC = < .001 | | | R^2^ Conditional = .06  R^2^ Marginal = .06  ΔR^2^ = .005, *p* = .01  ICC = < .001 | | | R^2^ Conditional = .06  R^2^ Marginal = .06  ΔR^2^ = < .001, *p* = .70  ICC = < .001 | | | |

**Table S25**. **Linear Mixed Model Analysis for Financial Benefits (N = 1453).**

|  | Model 1 | | | | Model 2 | | | | Model 3 | | | |
| --- | --- | --- | --- | --- | --- | --- | --- | --- | --- | --- | --- | --- |
|  | F | | df | *p* | F | | df | *p* | F | | df | *p* |
| Gender | 1.79 | | 1,1448 | .18 | 1.88 | | 1,1444 | .17 | 1.87 | | 1,1442 | .17 |
| Age | .26 | | 1,1447 | .61 | .29 | | 1,1444 | .59 | .28 | | 1,1442 | .60 |
| Utilisation Financial Benefits | 1.23 | | 1,1429 | .27 | .13 | | 1,1415 | .72 | .38 | | 1,1437 | .54 |
| Satisfaction Financial Benefits | 191.63 | | 1,1447 | < .001 | 130.86 | | 1,1443 | < .001 | 130.92 | | 1,1441 | < .001 |
| Utilisation Financial Benefits x Gender |  | |  |  | 2.36 | | 1,1444 | .13 | 1.86 | | 1,1441 | .17 |
| Utilisation Financial Benefits x Age |  | |  |  | .41 | | 1,1444 | .52 | .27 | | 1,1441 | .61 |
| Satisfaction Financial Benefits x Gender |  | |  |  | .63 | | 1,1443 | .43 | 1.10 | | 1,1441 | .30 |
| Satisfaction Financial Benefits x Age |  | |  |  | .11 | | 1,1443 | .74 | .15 | | 1,1441 | .70 |
| Utilisation Financial Benefits x Gender x Age |  | |  |  |  | |  |  | .003 | | 1,1441 | .96 |
| Satisfaction Financial Benefits x Gender x Age |  | |  |  |  | |  |  | .48 | | 1,1441 | .49 |
|  | | R^2^ Conditional = .20  R^2^ Marginal = .11  ICC = .11 | | | | R^2^ Conditional = .19  R^2^ Marginal = .11  ΔR^2^ = .005, *p* = .09  ICC = .10 | | | | R^2^ Conditional = .19  R^2^ Marginal = .11  ΔR^2^ = < .001, *p* = .71  ICC = .10 | | |

**Table S26**. **Linear Mixed Model Analysis for Socio/Cultural Benefits (N = 1224).**

|  | Model 1 | | | | Model 2 | | | | Model 3 | | | |
| --- | --- | --- | --- | --- | --- | --- | --- | --- | --- | --- | --- | --- |
|  | F | | df | *p* | F | | df | *p* | F | | df | *p* |
| Gender | 1.54 | | 1,1219 | .22 | 1.68 | | 1,1215 | .20 | 1.62 | | 1,1213 | .20 |
| Age | 1.43 | | 1,1219 | .23 | 1.22 | | 1,1215 | .27 | 1.36 | | 1,1213 | .24 |
| Utilisation Socio/Cultural Benefits | 16.24 | | 1,1219 | < .001 | 18.90 | | 1,1215 | < .001 | 17.79 | | 1,1213 | < .001 |
| Satisfaction Socio/Cultural Benefits | 25.77 | | 1,1219 | < .001 | 24.53 | | 1,1215 | < .001 | 20.00 | | 1,1213 | < .001 |
| Utilisation Socio/Cultural Benefits x Gender |  | |  |  | .01 | | 1,1215 | .91 | .01 | | 1,1213 | .93 |
| Utilisation Socio/Cultural Benefits x Age |  | |  |  | 4.76 | | 1,1215 | .03 | 4.45 | | 1,1213 | .04 |
| Satisfaction Socio/Cultural Benefits x Gender |  | |  |  | .03 | | 1,1215 | .86 | .33 | | 1,1213 | .57 |
| Satisfaction Socio/Cultural Benefits x Age |  | |  |  | 4.21 | | 1,1215 | .04 | 3.20 | | 1,1213 | .07 |
| Utilisation Socio/Cultural Benefits x Gender x Age |  | |  |  |  | |  |  | .32 | | 1,1213 | .57 |
| Satisfaction Socio/Cultural Benefits x Gender x Age |  | |  |  |  | |  |  | .36 | | 1,1213 | .55 |
|  | | R^2^ Conditional = .03  R^2^ Marginal = .03  ICC = .01 | | | | R^2^ Conditional = .03  R^2^ Marginal = .03  ΔR^2^ = .004, *p* = .28  ICC = < .001 | | | | R^2^ Conditional = .03  R^2^ Marginal = .03  ΔR^2^ = < .001, *p* = .83  ICC = < .001 | | |

**Table S27**. **Linear Mixed Model Analysis for Health/Safety Benefits (N = 1273).**

|  | Model 1 | | | | Model 2 | | | | Model 3 | | | |
| --- | --- | --- | --- | --- | --- | --- | --- | --- | --- | --- | --- | --- |
|  | F | | df | *p* | F | | df | *p* | F | | df | *p* |
| Gender | 1.05 | | 1,1267 | .31 | 1.28 | | 1,1263 | .26 | 1.00 | | 1,1261 | .32 |
| Age | < .001 | | 1,1268 | .99 | .004 | | 1,1264 | .95 | .01 | | 1,1262 | .92 |
| Utilisation Health/Safety Benefits | .14 | | 1,1261 | .71 | < .001 | | 1,1261 | .99 | 1.82 | | 1,1262 | .18 |
| Satisfaction Health/Safety Benefits | 56.66 | | 1,1267 | < .001 | 26.47 | | 1,1263 | < .001 | 23.73 | | 1,1261 | < .001 |
| Utilisation Health/Safety Benefits x Gender |  | |  |  | .92 | | 1,1263 | .34 | .01 | | 1,1261 | .92 |
| Utilisation Health/Safety Benefits x Age |  | |  |  | .13 | | 1,1262 | .71 | 1.17 | | 1,1260 | .28 |
| Satisfaction Health/Safety Benefits x Gender |  | |  |  | .42 | | 1,1262 | .52 | 1.50 | | 1,1260 | .22 |
| Satisfaction Health/Safety Benefits x Age |  | |  |  | 2.55 | | 1,1262 | .11 | 3.28 | | 1,1260 | .07 |
| Utilisation Health/Safety Benefits x Gender x Age |  | |  |  |  | |  |  | 4.34 | | 1,1261 | .04 |
| Satisfaction Health/Safety Benefits x Gender x Age |  | |  |  |  | |  |  | 1.27 | | 1,1260 | .26 |
|  | | R^2^ Conditional = .10  R^2^ Marginal = .04  ICC = .06 | | | | R^2^ Conditional = .10  R^2^ Marginal = .04  ΔR^2^ = .003, *p* = .47  ICC = .06 | | | | R^2^ Conditional = .10  R^2^ Marginal = .05  ΔR^2^ = .005 *p* = .04  ICC = .05 | | |

**Discussion**

Our preliminary findings reveal that women are generally less satisfied with their identity needs and their jobs compared to men. Despite this, women utilise work-life integration, financial, and socio-cultural benefits more than men, while men use health and safety benefits more. Additionally, individuals under 50 utilise work-life integration and health/safety benefits more than those over 50. Regarding satisfaction, women report higher satisfaction with financial and socio-cultural benefits compared to men.
